# Interleukin-1RA Mitigates SARS-CoV-2–Induced Inflammatory Lung Vascular Leakage and Mortality in Humanized K18-hACE-2 Mice

**DOI:** 10.1161/ATVBAHA.121.316925

**Published:** 2021-09-09

**Authors:** Shiqin Xiong, Lianghui Zhang, Justin M. Richner, Jake Class, Jalees Rehman, Asrar B. Malik

**Affiliations:** Department of Pharmacology and Regenerative Medicine and the Center for Lung and Vascular Biology (S.X., L.Z., J.R., A.B.M.), University of Illinois College of Medicine at Chicago.; Department of Microbiology and Immunology (J.M.R., J.C.), University of Illinois College of Medicine at Chicago.; Division of Cardiology, Department of Medicine (J.R.), University of Illinois College of Medicine at Chicago.; Now with Department of Cardiometabolic Diseases, Merck Research Laboratories, South San Francisco, CA (S.X.).

**Keywords:** edema, interleukin-1, pyroptosis, SARS-CoV-2, vascular system injur, ies

## Abstract

**Objective:**

SARS-CoV-2 (severe acute respiratory syndrome coronavirus 2) infection is a major cause of morbidity and mortality, often as a result of acute respiratory distress syndrome. Respiratory failure is characterized by a hyperinflammatory immune response, lung vascular injury, and edema formation. The potential for immunomodulatory therapy to prevent lung vascular injury and edema formation is not well understood.

**Approach and Results:**

We show that SARS-CoV-2 infection in humanized K18-hACE-2 mice activated inflammatory NLRP3 (NLR family pyrin domain containing 3)–caspase-1 pyroptotic signaling in lungs, release of IL (interleukin)-1β, and downregulation of the lung endothelial adherens junction protein VE (vascular endothelial)-cadherin. Primary human lung microvascular endothelial cells were susceptible to SARS-CoV-2 infection and displayed pyroptosis-like injury. We observed profound lung vascular injury post–SARS-CoV-2 infection and resultant protein-rich lung edema formation. Selective blockade of IL-1 receptor signaling by IL-1RA (IL-1 receptor antagonist) anakinra prevented downregulation of VE-cadherin, as well as accompanying lung vascular hyperpermeability. IL-1RA also significantly increased survival.

**Conclusions:**

These results provide insights into the central role of NLRP3–caspase-1 pyroptotic innate immune signaling and loss of lung endothelial adherens junctions in the mechanism of acute respiratory distress syndrome induced by SARS-CoV-2. Our data show that treatment with IL-1RA during activation of inflammasome provides the ideal scenario for preventing lung vascular injury and respiratory failure in coronavirus disease 2019 (COVID-19).

## Abstract 2

Supplemental Digital Content is available in the text.

**Highlights**

- Infection with SARS-CoV-2 (severe acute respiratory syndrome coronavirus 2) in the transgenic K18-hACE-2 mouse model results in NLRP3 (NLR family pyrin domain containing 3-caspase-1) inflammasome activation and IL (interleukin)-1β release in lungs.
- SARS-CoV-2 infection leads to downregulation of lung endothelial VE-cadherin and severe lung edema.
- Initiation of treatment with the IL-1 receptor antagonist anakinra after SARS-CoV-2 infection prevents VE (vascular endothelial)-cadherin downregulation and edema formation.
- Treatment with anakinra after SARS-CoV-2 infection reduces lung fibrosis and mortality.

The devastating coronavirus disease 2019 (COVID-19) pandemic is mediated by the SARS-CoV-2 (severe acute respiratory syndrome coronavirus 2) virus, which enters host cells via direct binding of the SARS-CoV-2 spike (S)-protein to the ACE-2 (angiotensin-converting enzyme 2) receptor and TMPRSS2 (transmembrane serine protease 2) membrane protease that are primarily expressed in type II lung alveolar epithelial cells.^1^ Although most COVID-19 patients have mild or moderate course of the disease, up to 5% to 10% of patients progress to acute respiratory distress syndrome (ARDS),^2^ characterized by maladaptive hyperinflammation, excessive influx of immune cells such as neutrophils in lungs, intractable hypoxemia due to lung endothelial hyperpermeability, and resultant fulminant protein-rich alveolar pulmonary edema, defective alveolar gas exchange, and respiratory failure.^3,4^ Analyses of postmortem lungs from COVID-19 patients and animal models show severe lung endothelial injury following the SARS-CoV-2 infection.^5–7^ Much of the focus on SARS-CoV-2–induced lung endothelial dysfunction has been on the coagulopathy,^5–7^ and less is known about the pathogenic role of the lung endothelium in promoting immune activation and lung edemagenesis.

Hyperinflammation and cytokine storm are key pathogenic features underlying severe COVID-19 mediating ARDS and respiratory failure.^8–12^ While >90% of COVID-19 patients exhibit mild-to-moderate symptoms because their host-defense responses efficiently to eliminate the virus after which inflammation subsides, a subset develops an exaggerated feed-forward inflammatory response in which immune cells release excessive cytokines and chemokines, resulting in damage of lungs and other tissues.^10–12^ Upregulation of NLRP3 (NLR family pyrin domain containing 3-caspase-1) inflammasome, which activates the inflammatory caspase-1 required for the cleavage and release of the proinflammatory cytokine IL (interleukin)-1β, has recently been identified as a critical prognostic marker of poor COVID-19 outcome in patients^13,14^ and mouse and cell culture studies.^15^ SARS-CoV-2 infection also induces inflammasome activation and pyroptosis in human primary monocytes.^16^ The importance of inflammasome activation for COVID-19 severity is likely a reflection of the central pathogenic role of the NLRP3 inflammasome and IL-1β generation in ARDS. In ARDS induced by endotoxemia or bacterial sepsis, IL-1β disrupted lung endothelial barrier by downregulating the transcription factor CREB (cAMP response element-binding protein) and its target VE (vascular endothelial)-cadherin—the primary adhesive protein mediating homotypic endothelial cell interaction in microvessels at adherens junctions.^17^ In addition, IL-1β induced recruitment of neutrophils through enhanced migration across the leaky endothelial barrier, contributing to the hyperinflammation state.^17^ The lung endothelium is not only a target of IL-1β released by immune cells but endothelial cells themselves can release IL-1β while undergoing pyroptosis—a form of inflammatory cell death.^18^

The rapid development of COVID-19 vaccines with efficacy rates of 70% to 95% has been highly successful in reducing COVID-19 transmission and prevalence; however, vaccine hesitancy, limited vaccine accessibility in poor countries, loss of vaccine efficacy, and emergence of novel SARS-CoV-2 variants of concern showing greater transmissibility^19–22^ underscore the urgent need for other therapeutic approaches for severe COVID-19. Therapies targeting severe COVID-19 can be broadly divided into 2 categories: antivirals, which prevent cell entry or replication of SARS-CoV-2, or immunomodulatory agents, which arrest the vicious cycle of hyperinflammation.^2,23^ While broad immunomodulators such as dexamethasone show success in patients with severe COVID-19 requiring mechanical ventilation,^24^ these immunosuppressive approaches also raise concerns by compromising host-defense responses required to eliminate SARS-CoV-2^25,26^. We posited in light of the critical role of IL-1β in disrupting lung endothelial barrier function during ARDS,^17^ a therapy targeting SARS-CoV-2 activated inflammasome–caspase-1/11–IL-1β signaling might avert lung vascular endothelial injury and edema formation and subsequent respiratory failure. Anakinra—a minimally modified IL-1RA (IL-1 receptor antagonist), which has been approved by the US Food and Drug Administration for patients with autoimmune disease,^27^ may block IL-1β signaling and prevent the lung complications of SARS-CoV-2. Here, we used the humanized K18-hACE-2 mouse model in which lung alveolar type II epithelium expressed human ACE-2 and hence could be infected by SARS-CoV-2.^28–31^

## Materials and Methods

The data that support the findings of this study will be available from the corresponding author upon request.

### Mice

Hemizygous K18-hACE c57BL/6J mice (strain#034860: B6.Cg-Tg(K18-ACE-2)2Prlmn/J) were purchased from The Jackson Laboratory. Mice of different ages and both sexes were intranasally inoculated with either lethal dose (1×10^5^ p.f.u. [plaque-forming unit]), sublethal dose (2×10^4^ p.f.u.) of SARS-CoV-2, or mock-infected with PBS as controls. Virus inoculations were performed under anesthesia that was induced and maintained with ketamine hydrochloride and xylazine, and all efforts were made to minimize animal suffering. Biosafety level 3 experiments employing live SARS-CoV-2 were performed by personnel equipped with powered air-purifying respirators in strict compliance with the National Institutes of Health guidelines and approved by the University of Illinois Animal Care and Use Committee and the University of Illinois Institutional Biosafety Committee.

Genotyping of mice was performed by polymerase chain reaction (PCR) using tail DNA. All experimental mice were 2 to 3 months old. For the number of animals needed to achieve statistically significant results, we conducted the a priori power analysis. We calculated power and sample sizes according to data from small pilot experiments, variations within each group of data, and variance similarities between the groups that were statistically compared. Animals with sex- and age-matched littermates were randomly included in experiments. No animals were excluded attributed to illness after experiments. Animal experiments were performed in a blinded fashion.

### Cells and Viruses

The 2019n-CoV/USA_WA1/2019 isolate of SARS-CoV-2 (NR-52281) was obtained from BEI Resources (National Institute of Allergy and Infectious Diseases, National Institutes of Health). Vero E6 (CRL-1586; American Type Culture Collection) cells cultured at 37 °C in DMEM were supplemented with 10% fetal bovine serum, 10 mM HEPES (pH 7.3), 1 mM sodium pyruvate, 1× nonessential amino acids, and penicillin-streptomycin. Infectious stocks were grown by inoculating Vero E6 cells and collecting supernatant upon observation of cytopathic effect; debris were removed by centrifugation and passage through a 0.22-μm filter. Supernatant was then aliquoted and stored at −80 °C. Titers of viral stocks (5×106 p.f.u.) were determined by plaque assays. Primary human lung microvascular endothelial cells (hLMVECs) from Lonza were cultured in EBM-2 supplemented with 10% endotoxin-free fetal bovine serum (Omega Scientific). Human lung carcinoma epithelial cells (A549) with hACE-2 (human angiotensin-converting enzyme 2) stably expressed (NR-53821) were obtained from BEI Resources. Virus propagation experiments were performed under biosafety level 3 containment with approval of the University of Illinois College of Medicine Institutional Biosafety Committee.

### Antibodies and Reagents

We purchased antibodies against NLRP3 (AdipoGen; AG-20B-0014-C100), caspase-1 (AdipoGen; AG-20B-0042-C100), caspase-11 (Novus Biologicals; NB-120-10454), IL-1β (R&D Systems; AF-401-NA), caspase-4 (Santa Cruz Biotechnology, Inc; sc-56056), caspase-5 (Santa Cruz Biotechnology, Inc; sc-393346), CREB (Cell Signaling; 48H2), VE-cadherin (Santa Cruz Biotechnology; sc-6458), and β-actin (Sigma; A-5316). Polyclonal anti–SARS-CoV-2 spike glycoprotein was obtained from BEI Resources. IL-1R antagonist anakinra (IL-1RA, #407616) was obtained from Calbiochem; anakinra (C_759_H_1186_N_208_O_232_S_10._) is a recombinant biopharmaceutical and slightly modified version of the human IL-1RA. Albumin (catalog A7906), O-dianisidine dyhydrochloride (catalog D3252), and protease inhibitor cocktail (catalog P8340) were from Millipore Sigma. The CytoTox 96 Non-Radioactive Cytotoxicity Assay kit (G1780) was purchased from Promega. Enhanced chemiluminescence Western blotting detection reagents and nitrocellulose membranes (Hybond ECL) were from Amersham Biosciences Corp. Lipofectamine 3000 transfection reagents were from Invitrogen.

### mRNA Expression by RT-PCR and Quantitative Real-Time PCR

One-step RT-PCR (reverse transcription polymerase chain reaction) amplification was performed using the SuperScript 1-step RT-PCR system with Platinum Taq DNA polymerase (Invitrogen). One microgram total RNA isolated from hACE-2–stabled expressed A549, A549, and primary hLMVECs was used as a template for subsequent 1-step RT-PCR. One-step RT-PCR products were analyzed by electrophoresis on 1.2% agarose gels containing ethidium bromide. For quantitative real-time PCR, total RNA (1 μg) was reverse transcribed with Superscript III (Invitrogen) using random primers. Synthesized cDNA samples were amplified in the ABI PRISM 7000 Sequence Detection System (Applied Biosystems) thermocycler using SYBR Green JumpStart Taq ReadyMix (MilliporeSigma). The results of relative expression were normalized to GAPDH mRNA levels in each sample. Results are expressed as mean±SEM.

### Western Blotting

Lungs of mice post–SARS-CoV-2 infection and shACE-2 V2.4 treatment were surgically removed and washed in cold PBS and homogenized on ice in lysis buffer (50 mmol/L HEPES [pH 7.4], 50 mmol/L NaCl, 1% Triton X-100, 5 mmol/L EDTA, 1 mmol/L DTT, 1 mmol/L PMSF, 10 μg/mL aprotinin, 10 μg/mL leupeptin, 10 mmol/L sodium pyrophosphate, 50 mmol/L sodium fluoride, and 1 mmol/L sodium orthovanadate) for 1 hour. Samples were centrifuged at 12 000*g* for 10 minutes. Supernatants were collected for measurement of protein concentrations by BCA (bicinchoninic acid) methods. Samples were mixed with sample buffer and boiled in a heat blocker at 90 °C for 10 minutes to ensure inactivation of the remaining live virus before moving out of the biosafety level 3 facility. Boiled samples were separated by SDS-PAGE, transferred to nitrocellulose membranes, and incubated overnight with the indicated antibodies. After incubation with secondary antibodies, proteins were detected by enhanced chemiluminescence. Quantification of band intensities by densitometry was carried out using the ImageJ software.

### Histology

Mouse lungs were collected in a hood within animal biosafety level 3 facility. Lung tissues post-PBS reperfusion were grossly examined and then fixed in 10% formalin solution, and paraffin sections (5 μm in thickness) were prepared routinely. Hematoxylin and eosin and the modified Masson trichrome stains were used to identify histopathologic changes. Quantification of lung fibrosis was assessed according to the Ashcroft method of analysis with a standardized modification as described previously.^32–34^ The histopathology of the lung tissue was evaluated by light microscopy. Aperio bright-field 20× whole slide scans were performed. Infiltration of activated polymorphonuclear neutrophils into the lung post–SARS-CoV-2 infection was calculated by a computer-based stereological method described previously.^35,36^

### MPO Assay

Lung tissues isolated from mice post–SARS-CoV-2 infection and drug treatment were homogenized in cold lysis buffer (50 mmol/L HEPES [pH 7.4], 50 mmol/L NaCl, 1% Triton X-100, 5 mmol/L EDTA, 1 mmol/L DTT, 10 mmol/L sodium pyrophosphate, 50 mmol/L sodium fluoride, and 1 mmol/L sodium orthovanadate) with freshly added proteinase inhibitors. Samples were centrifuged at 13 000*g* for 10 minutes at 4 °C to remove insoluble material. Protein concentration (about 1–2 µg/µL) was determined by the standard Bio-Rad BCA method. The supernatants (protein samples) were collected for a direct MPO (myeloperoxidase) assay. MPO reaction was performed according to Kit’s instructions. Reaction was stopped by adding 100 µL of stop buffer (2 M HCL or 1 M H_2_SO_4_) for 5 minutes. Absorption was measured at 460 nm to estimate MPO activity. Data were calculated as A_460_·min^−1^·g^−1^ protein.

### Lung Vascular Endothelial Permeability Measurements

Evans Blue–albumin pulmonary transvascular flux measurements were performed to measure lung vascular leakage. Briefly, 200 μL Evans Blue–albumin (1% Evans Blue dye [EBD] and 4% albumin in PBS) was injected into anesthetized mice and allowed to circulate in the blood vessels for 30 minutes. Mice were euthanized, and lungs were perfused by 5 mL PBS. Lung tissues were then excised, weighed, homogenized in 1 mL PBS and extracted overnight in 2 mL formamide at 60 °C. Samples were centrifuged at 10 000*g* for 5 minutes. Evans Blue concentration in lung homogenate supernatants was quantified by the spectrophotometric method at an absorbance of 620 nm. Tissue EBD content (μg EBD/g fresh lung tissue) was calculated by comparing tissue supernatant A_620_ readings with an EBD standard curve. Concentration of EBD was determined in micrograms per gram of wet lung tissue. The ratio of wet lung to dry lung weight for edema measurement was calculated.

### Evaluation of Endothelial Pyroptosis

Release of LDH (lactate dehydrogenase) from pyroptotic cells was measured by supplying lactate, NAD^+^, and INT (tetrazolium salt) as substrates in the presence of diaphorase. LDH release method with a combination of inflammatory caspase-1 or 11 activation has been used for evaluation of pyroptotic cell death.^18,37,38^ Briefly, 80% confluent hLMVECs were seeded in 24-well cell culture plate. Cells culturing in serum-free EBM-2 medium were infected with increasing titers of SARS-CoV-2 for 1 day. The supernatants were collected and centrifuged (500*g*, 5 minutes). Then 50 µL of the supernatant from each sample was transferred to a new 96-well plate and mixed with 50 µL of the CytoTox 96 Reagent for 30 minutes at room temperature. Stop Solution (50 μL) was added to each well of the 96-well plate. Serum-free medium was used as the 0% control, and lysates of the untreated cell were used as the 100% maximal release. The absorbance was measured at 490 nm within 1 hour after adding the Stop Solution on a spectrophotometric microplate reader.

### Statistical Analysis

Data were analyzed by 2-tailed unpaired Student *t* test for comparisons of 2 groups or 1-way ANOVA of the repeated experiments followed by the Tukey post hoc pairwise multiple comparisons when appropriate with Prism 9 (GraphPad Software, Inc). *P* of <0.05 was considered significant. For all bar graphs, mean±SEM is plotted unless otherwise indicated. Normality and variance were not tested to determine whether the applied parametric test was appropriate.

## Results

SARS-CoV-2 infection induces NLRP3–caspase-1 inflammatory signaling, IL-1β release, VE-cadherin downregulation, and polymorphonuclear neutrophil infiltration in K18-hACE-2 mice.

**Figure 1.**
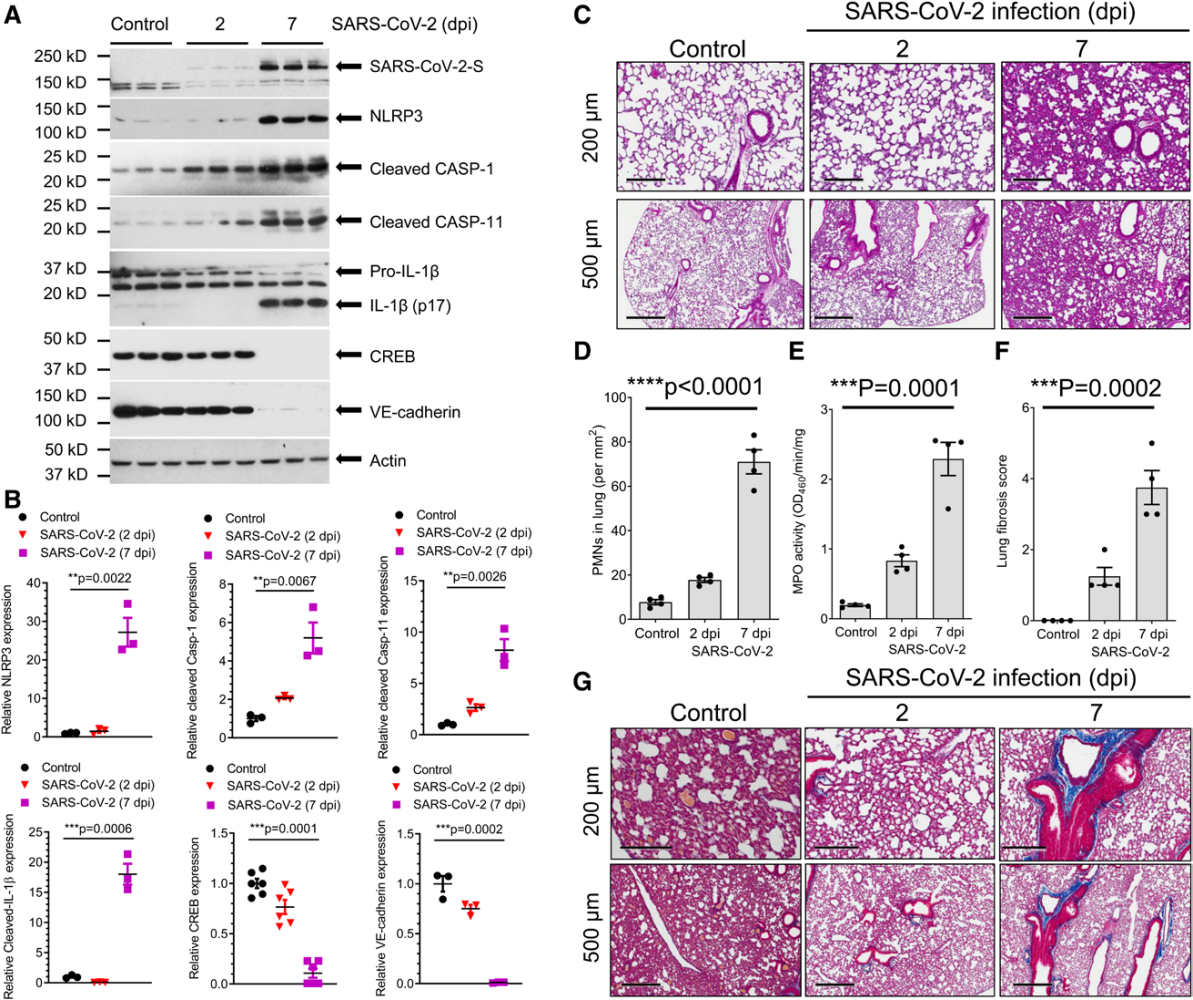
**SARS-CoV-2 infection activates lung NLRP3–CASP (NLR family pyrin domain containing 3-caspase)-1 inflammatory signaling and lung inflammation in K18-ACE-2 mice.** K18-hACE-2 humanized mice (2 mo old) were inoculated with SARS-CoV-2 (severe acute respiratory syndrome coronavirus 2; 1×10^5^ p.f.u.) for 2 and 7 d. **A**, Expression of NLRP3 inflammasome, cleavage of CASP-1/11, IL (interleukin)-1β maturation, and CREB (cAMP response element-binding protein) and VE (vascular endothelial)-cadherin expression in the lung following SARS-CoV-2 infection at day 7 as assessed by immunoblotting with quantification in **B**. Two-tailed unpaired *t* test. **C**, Lung histopathology in K18-hACE-2 mice post-inoculation. Hematoxylin and eosin staining showed inflammatory infiltrates composed of lymphocytes and neutrophils. Representative images with lower power magnification (scale bars, 500 µm) and higher power magnification (scale bars, 200 µm) from 2 independent experiments are shown. **D**, Morphometric quantification of neutrophil infiltration in lungs (n=4). ****P<0.0001, 2-tailed unpaired *t* test. **E**, Analysis of neutrophil infiltration by measurement of lung tissue MPO (myeloperoxidase) activity (n=4). Two-tailed unpaired *t* test. Polymorphonuclear neutrophil (PMN) infiltration primarily occurred between days 2 and 7 (**D** and **E**). **F**, Lung fibrosis was half-quantified using the Ashcroft method of analysis. Results are shown as mean±SEM. ***P<0.001, 2-tailed unpaired *t* test. **G**, Lung collagen deposition evaluated by the Masson trichrome stain. Collagen fibers are evident on day 7. Representative images are selected from 2 independent experiments (n=4 mice per group). dpi indicates days post inoculation.

We first investigated changes in NLRP3 inflammasome and expression of VE-cadherin in humanized K18-hACE-2 mice challenged with SARS-CoV-2; the epithelial cell K18 (cytokeratin-18) promoter in these mice induces expression of hACE-2, and thus epithelial cells can be infected by SARS-CoV-2.^28–31^ In these mice, nonepithelial cells express murine ACE-2, and, therefore, they are not infected by the virus. Lung SARS-CoV-2 infection and replication were evaluated by immunoblotting for the spike protein in total lung tissue. We found that intranasal inoculation with SARS-CoV-2 (strain 2019n-CoV/USA_WA1/2020) upregulated NLRP3, inflammatory caspase-1 and -11, and induced maturation of IL-1β, while concomitantly downregulating endothelial adherens junction VE-cadherin expression (Figure 1A and 1B). A previous study identified the critical role of transcription factor CREB in regulating the expression of VE-cadherin.^17^ The post–SARS-CoV-2 lungs also showed increased polymorphonuclear neutrophil infiltration into lung (Figure 1C through 1E). At 7 days post inoculation (dpi), we observed marked deposition of collagen fibers (Figure 1F and 1G) suggesting onset of lung fibrosis. These findings show that even though SARS-CoV-2 can only infect lung epithelial cells in K18-hACE-2 mice, it also activates NLRP3 inflammatory signaling resulting in the release of cytokine IL-1β, which may suppress VE-cadherin in neighboring noninfected endothelial cells, mirroring what has been reported for VE-cadherin downregulation in bacterial infections.^17^

### SARS-CoV-2 Infection Induces Lung Vascular Hyperpermeability and Edema Formation

**Figure 2.**
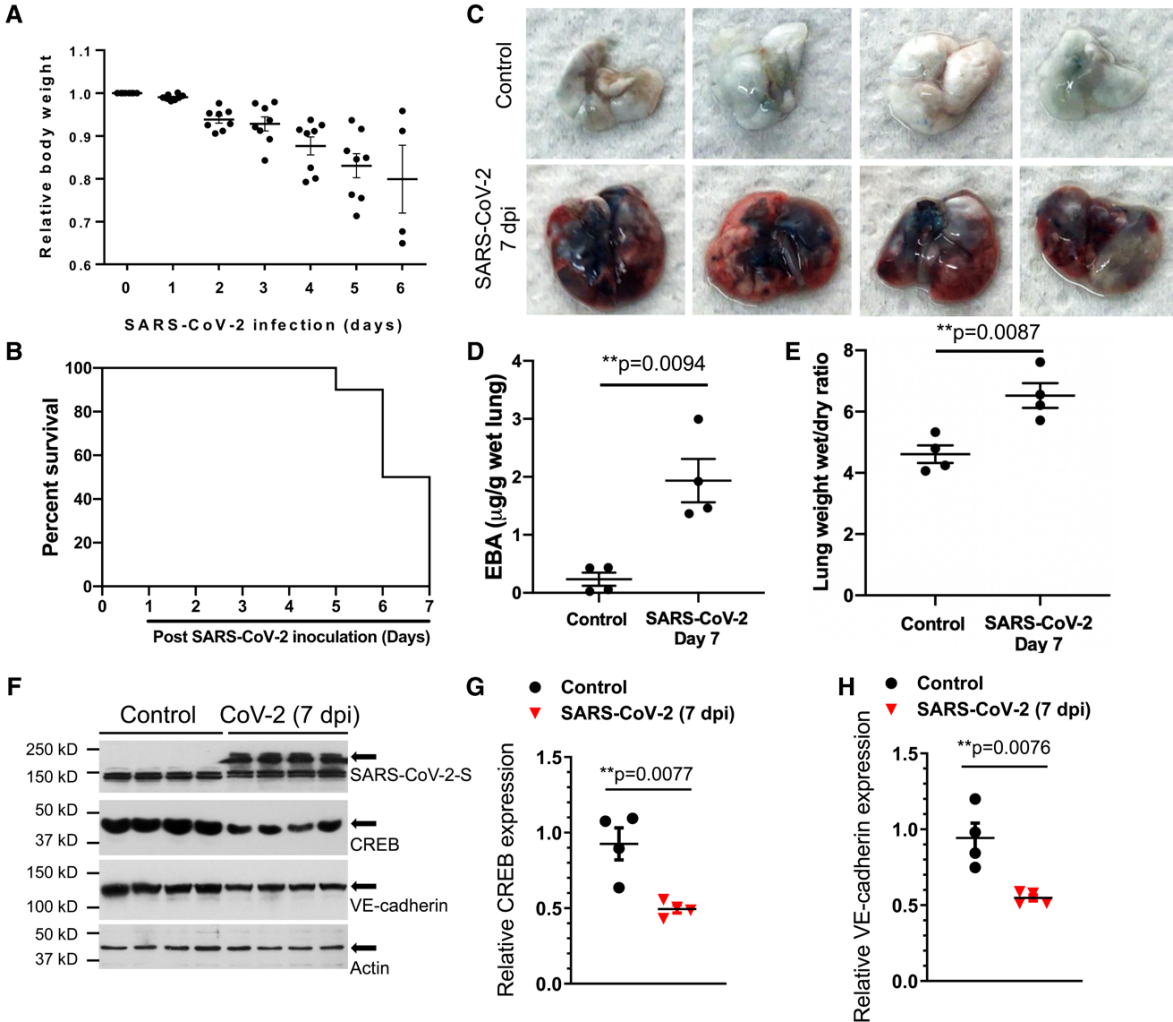
**SARS-CoV-2 infection induces lung vascular hyperpermeability and reduces CREB and VE-cadherin expression in K18-ACE-2 mice.** K18-hACE-2 mice (2 mo old) were infected with SARS-CoV-2 (severe acute respiratory syndrome coronavirus 2; 1×10^5^ p.f.u.) for the indicated days. **A**, Mouse body weight was measured post–SARS-CoV-2 inoculation. **B**, Survival of mice post–SARS-CoV-2 infection for the indicated days was monitored and is presented as a Kaplan-Meier plot. **C**, Lung vascular leak assessed by Evans Blue albumin (EBA) dye infusion is shown in representative images from 2 independent experiments. **D**, Quantification of lung vascular permeability (n=4). Extracted dye contents were quantified by measuring absorption at 620 nm. **E**, Lung edema formation by the ratio of the wet lung to dry lung weight was assessed (n=4). Two-tailed unpaired *t* test. **F–H**, CREB (cAMP response element-binding protein) and VE (vascular endothelial)-cadherin were assessed by immunoblotting. Quantification of protein expression was analyzed by ImageJ. Results are shown as mean±SEM. **P<0.01, 2-tailed unpaired *t* test (n=4). dpi indicates days post inoculation.

Following SARS-CoV-2 infection (1×10^5^ p.f.u.), K18-hACE-2 mice showed progressive body weight loss and high mortality by day 7 (Figure 2A and 2B). As IL-1β–induced downregulation of VE-cadherin expression mediates lung vascular injury in bacterial sepsis via suppression of the transcription factor CREB,^17^ we addressed the possibility that SARS-CoV-2 infection may also lead to lung hyperpermeability of the endothelium via disruption of the lung endothelial barrier due to downregulation of VE-cadherin expression. We observed that K18-hACE-2 mice challenged with SARS-CoV-2 showed marked increases in lung vascular permeability and severe pulmonary edema that are the central features of ARDS (Figure 2C through 2E). Furthermore, SARS-CoV-2 infection at 7 dpi reduced both CREB and VE-cadherin expression (Figure 2F through 2H) consistent with the described role of SARS-CoV-2 infection impairing adherens junctions.

### SARS-CoV-2 Infection in Human Microvascular Endothelial Cells Induces Pyroptotic Cell Death Coincident With Activation of NLRP3–Caspase-1 Signaling

**Figure 3.**
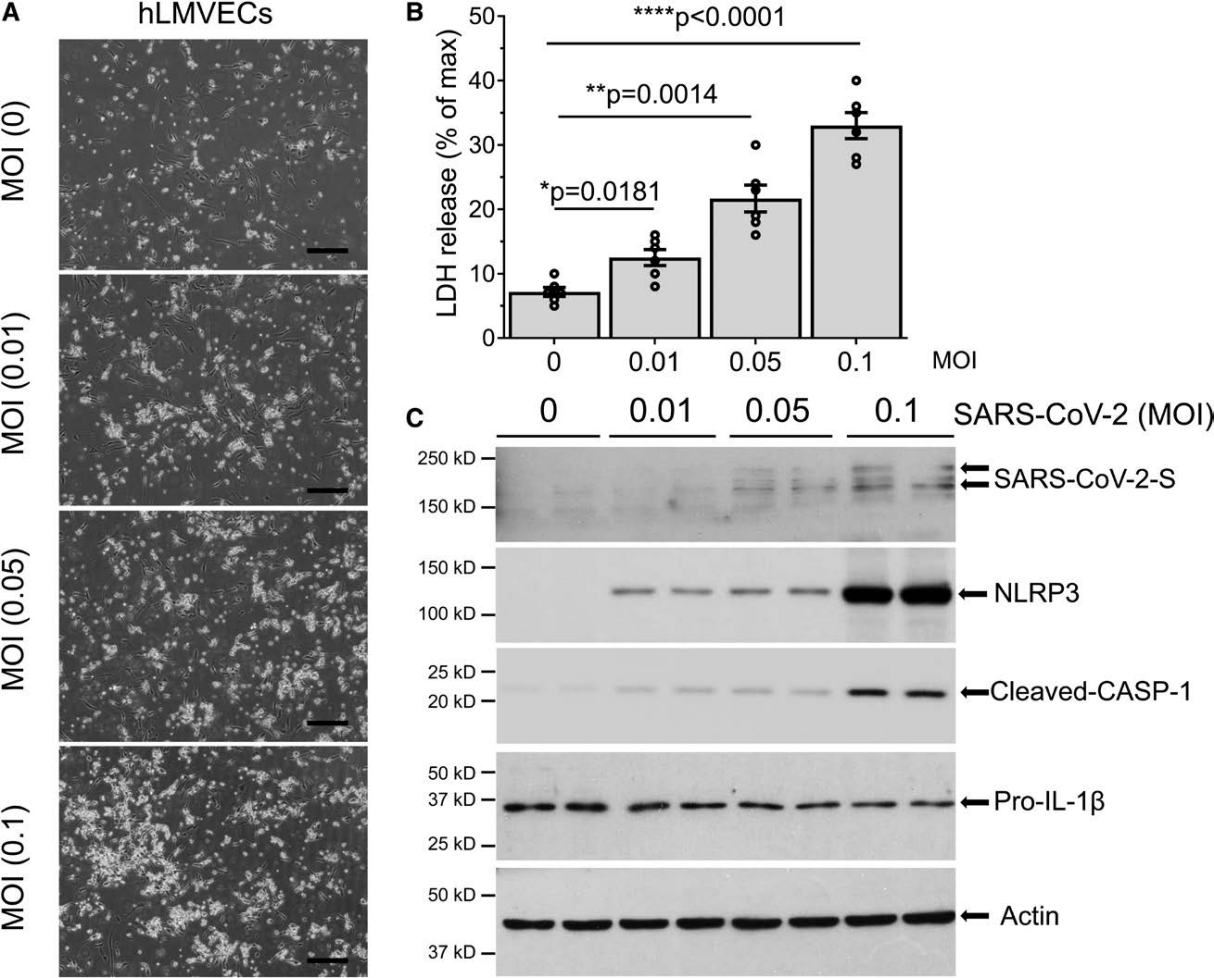
**SARS-CoV-2 (severe acute respiratory syndrome coronavirus 2) infection induces human lung microvascular endothelial cells (hLMVECs) pyroptosis coincident with activation of inflammatory NLPR3–CASP (NLR family pyrin domain containing 3-caspase)-1 signaling and IL (interleukin)-1β cleavage.** hLMVECs were infected with increasing titers of SARS-CoV-2 (MOI [multiplicity of infection]: 0.01, 0.1, and 0.5) for 1 d. **A**, Phase-contrast micrographs of hLMVECs post–SARS-CoV-2 infection for 1 d. Scale bars, 200 µm. **B**, Cytotoxicity activity of SARS-CoV-2 infection was analyzed by LDH (lactate dehydrogenase) release. n=6/group. *P<0.05, **P<0.01, ****P<0.0001, 2-tailed unpaired *t* test. **C**, Western blot was performed with the indicated antibodies. Activation of inflammatory NLRP3–CASP-1 pyroptotic signaling and IL-1β cleavage were shown in the blots.

Human pathology and autopsy studies showed that lung vascular endothelium is a primary site of injury in SARS-CoV-2 infection.^5,6,39–41^ To address mechanisms of endothelial injury, we determined endogenous mRNA expression of the SARS-CoV-2 receptor ACE-2 and the associated main protease, TMPRSS2, in hLMVECs. mRNA expression of ACE-2 and TMPRSS2 was evident in both A549 alveolar epithelial type II cells and hLMVECs (Figure S1 in the Data Supplement), suggesting that alveolar epithelial type II cells and lung microvascular endothelial cells exhibit similar susceptibility to SARS-CoV-2 infection. hLMVECs exposed to increasing titers of SARS-CoV-2 infection for 1 day displayed dose-dependent increase in cell death (Figure 3A and 3B). The observed cell death of hLMVECs at 1 dpi was quantified by LDH release and was consistent with pyroptotic cell death, as evident by the activation of inflammatory NLRP3–caspase-1 signaling and maturation of IL-1β (Figure 3C). We also observed that at high titers of SARS-CoV-2, hLMVECs became susceptible to infection as seen in the endothelial expression of the SARS-CoV-2 spike protein (Figure 3C). Our data thus support a key role of lung vascular endothelium in responding to SARS-CoV-2 infection.

### Blockade of IL-1 Receptor Prevents SARS-CoV-2–Induced Lung Endothelial Barrier Injury

**Figure 4.**
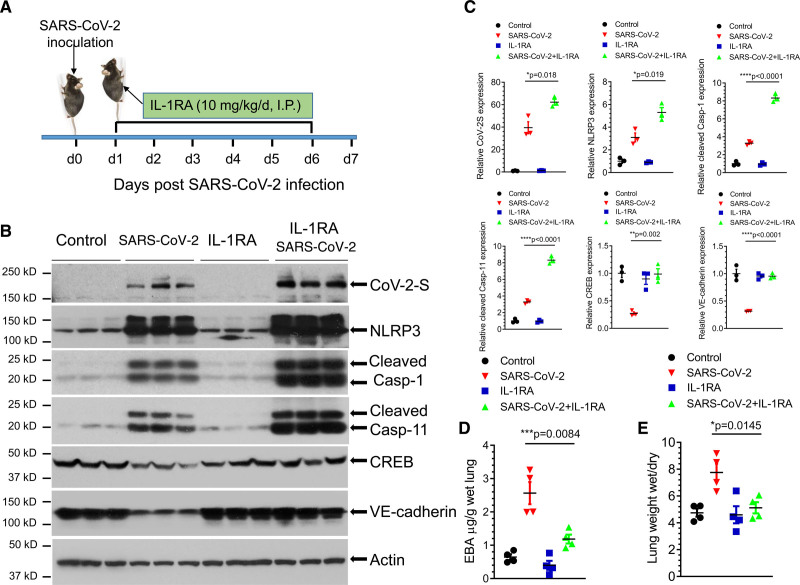
**IL (interleukin)-1 receptor blockade by IL-1 receptor antagonist anakinra prevents SARS-CoV-2 (severe acute respiratory syndrome coronavirus 2)–induced lung vascular hyperpermeability and edema.** K18-hACE-2 mice (2 mo old) were infected with a sublethal dose of SARS-CoV-2 (2×10^4^ p.f.u.) for 7 d. **A**, Mice were administered IL-1RA (IL-1 receptor antagonist) anakinra (10 mg/kg per d) or vehicle by IP (intraperitoneal) injection at 24 h post-infection and every day thereafter as shown. **B** and **C**, Lung lysates were assessed for the expression of SARS-CoV-2 spike protein, NLRP3 (NLR family pyrin domain containing 3), cleaved Casp (caspase)-1 and Casp-11, CREB (cAMP response element-binding protein), and VE (vascular endothelial)-cadherin by immunoblotting. IL-RA did not prevent the infection-induced expression of the viral spike protein or inflammasome activation but restored the expression of CREB and VE-cadherin. Protein expression levels from blots are quantified in **C**. Two-tailed unpaired *t* test (n=3). *P<0.05, **P<0.01 ****P<0.0001. **D**, Lung vascular permeability was determined by lung transvascular albumin flux measurements using Evans Blue albumin (EBA). **E**, Lung edema was determined by ratio of wet-to-dry lung weights (n=4 in each group). Results are shown as mean±SEM. *P<0.05, ***P<0.001.

Lung vascular hyperpermeability is the primary cause of protein-rich edema formation in lungs leading to ARDS and ultimately to death due to defective gas exchange in the fluid-filled alveoli.^3^ To address whether IL-1β–induced downregulation of VE-cadherin could underpin lung vascular hyperpermeability observed in SARS-CoV-2 infection in mice, we investigated the therapeutic potential of the IL-1RA anakinra.^27^ K18-hACE-2 mice received anakinra (10 mg/kg per day, IP [intraperitoneal]) 24 hours after the sublethal dose of SARS-CoV-2 (2×10^4^ p.f.u.) and thereafter, daily injections (Figure 4A). We observed that IL-1RA abrogated the downregulation of CREB and VE-cadherin expression even though blockade of IL-1R signaling by IL-1RA did not prevent the upstream activation of the NLRP3–caspase-1 inflammasome pathway and the lung became infected with SARS-CoV-2 (Figure 4B and 4C). IL-1R blockade prevented lung vascular leakage (Figure 4D) and edema formation (Figure 4E) indicating the primary role of IL-1 in mediating lung vascular endothelial injury and edema. Together, these data show that IL-1 receptor antagonism acts downstream of NLRP3 inflammasome and restores expression of lung endothelial adherens junction protein VE-cadherin and thereby prevents lung vascular hyperpermeability in SARS-CoV-2 infection.

### IL-1 Receptor Antagonism Mitigates Lung Neutrophil Infiltration, Lung Fibrosis, and Mortality in Mice Induced by High Titers of SARS-CoV-2

**Figure 5.**
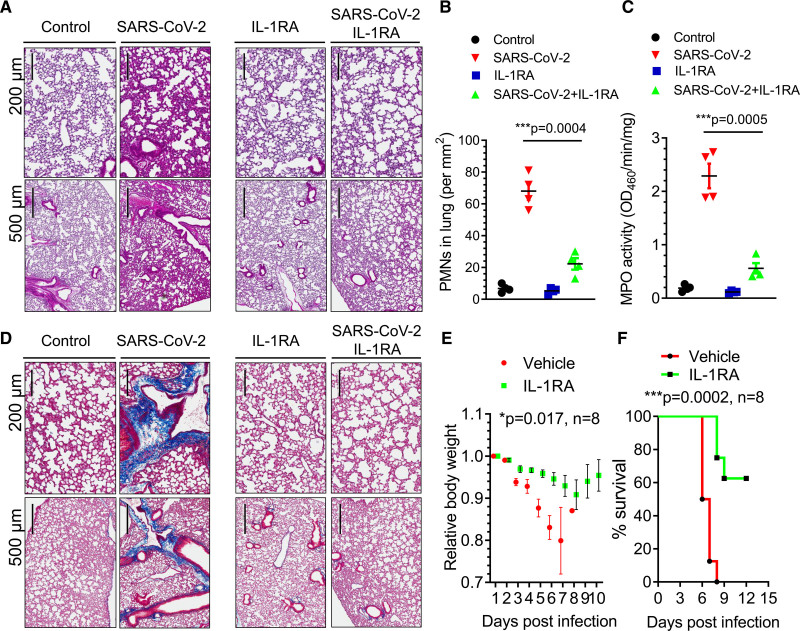
**IL (interleukin)-1 receptor blockade mitigates SARS-CoV-2 (severe acute respiratory syndrome coronavirus 2)–induced acute respiratory distress syndrome, fibrosis, and mortality in K18-ACE-2 mice.** K18-hACE-2 mice (2 mo old) were infected with a lethal dose of SARS-CoV-2 (1×10^5^ p.f.u.). Mice also received the IL-1RA (IL-1 receptor antagonist) anakinra (10 mg/kg per d) or vehicle by IP (intraperitoneal) injection at 24 h post-infection and daily thereafter. **A**, Lung histopathology in K18-hACE-2 mice post-inoculation. Hematoxylin and eosin–stained sections (scale bars, 200 and 500 µm) showed inflammatory infiltrates composed of lymphocytes and polymorphonuclear neutrophils (PMNs). Representative images from 2 independent experiments are shown. **B**, PMN numbers in lung were morphometrically quantified (n=4). Two-tailed unpaired *t* test (n=4). ***P<0.001. **C**, Lung PMN infiltration determined by measurement of lung tissue MPO (myeloperoxidase) activity (n=4). Two-tailed unpaired *t* test (n=4). ***P<0.001. **D**, Lung collagen deposition (blue) post–SARS-CoV-2 infection and the effects of IL-1RA treatment evaluated by Masson trichrome staining. Representative images from 2 independent experiments are shown (scale bars, 200 and 500 µm). **E**, Body weight of mice was monitored post–SARS-CoV-2 inoculation. IL-1RA treatment prevented weight loss. Two-tailed unpaired *t* test (n=8). *P<0.05. **F**, Survival of mice post–SARS-CoV-2 infection presented as a Kaplan-Meier plot. IL-RA treatment markedly improved survival. ***P<0.001. n=8.

We next investigated the effects of IL-1RA in mice challenged with a lethal dose of SARS-CoV-2 (1×10^5^ p.f.u.). K18-hACE-2 mice received IL-1RA (10 mg/kg per day) or vehicle by IP injection at 24 hours post–SARS-CoV-2 and daily injections thereafter. We observed that IL-1RA therapy prevented neutrophil infiltration in infected lungs (Figure 5A through 5C). Interestingly, lung collagen fiber deposition at 7 dpi was also significantly reduced by IL-1RA (Figure 5D; Figure II in the Data Supplement). The severe and progressive loss of body weight was suppressed with IL-1RA treatment (Figure 5E). Strikingly, 90% of the mice in the SARS-CoV-2–infected group died at 7 dpi, whereas 60% mice treated with IL-1RA were still alive at 12 dpi (Figure 5F). Thus, IL-1RA anakinra markedly reduced mortality in a lethal model of SARS-CoV-2 infection.

## Discussion

Although several animal models have been developed to investigate the susceptibility to SARS-CoV-2 infection, few of them have recapitulated the severe disease seen in humans who have been hospitalized.^29,42^ The development of countermeasures that reduce COVID-19 morbidity and mortality is a priority for the global research community, and animal models are particularly urgent for this effort. Hamsters, ferrets, and nonhuman primates develop mild-to-moderate viral disease and recover spontaneously.^29,43,44^ Conventional laboratory strains of mice cannot be infected efficiently with SARS-CoV-2 because hACE-2, but not mouse ACE-2, supports SARS-CoV-2 binding.^45,46^ Multiple strategies for introducing hACE-2 into mice have been developed, including (1) transient introduction of hACE-2 via adenoviral or adenoviral-associated vectors^47,48^; (2) expression of hACE-2 as a transgene driven by heterologous gene promoters^28,49,50^; or (3) expression of hACE-2 by the mouse ACE-2 promoter.^51,52^ While these animals all support SARS-CoV-2 infection, only the models with hACE-2 expression driven by the HFH4 (hepatocyte nuclear factor-3/forkhead homologue 4) promoter^50^ and the epithelial cell K18 promoter develop severe disease and show high mortality.^28,31,53,54^ Rathnasinghe et al^55^ recently demonstrated that the K18-hACE-2 model is more stringent for testing vaccines and antivirals than the adenovirus delivery system. In the current study, we used K18-hACE-2 mice to investigate the role of inflammasome activation in triggering lung vascular leakage and address the role of IL-1RA in ameliorating SARS-CoV-2–induced lung vascular permeability, lung edema formation, and mortality. Our findings do not rule out the need for additional animal models such as nonhuman primates to better understand the mechanisms of the devastating systemic inflammatory syndrome and inflammatory lung injury but provide proof of concept in a highly reproducible mouse model of COVID-19–induced lung injury and failure.

Multiple small clinical trials have investigated the role of immunomodulation in COVID-19 patients because the hyperinflammatory state is a key pathogenic factor of disease initiation and progression. Recently, 2 major trials involving the IL-6 antagonists tocilizumab and sarilumab were conducted in COVID-19 patients. However, the conclusions were contradictory.^56–58^ In the REMAP-CAP trial (A Randomised, Embedded, Multi-Factorial, Adaptive Platform Trial for Community-Acquired Pneumonia), critically ill COVID-19 patients receiving the IL-6 receptor antagonists tocilizumab and sarilumab showed improved survival.^56^ However, in noncritically ill hospitalized patients,^57^ tocilizumab did not improve survival. These findings suggest that selection of the target patient population may be critical for the success of immunomodulatory therapy in COVID-19 patients.

In the present study, we first focused on IL-1 receptor antagonism instead of IL-6 antagonism because IL-1β signaling initiates the breakdown of the lung vascular barrier in ARDS.^17^ We found that the K18-hACE-2 mouse accurately modeled severe lung hyperpermeability following SARS-CoV-2 infection, similar to advanced COVID-19 patients. Furthermore, we observed that anakinra treatment restored the expression of the endothelial adherens junction protein VE-cadherin, prevented lung hyperpermeability, and reduced mortality. Results from clinical trials with anakinra in a small number of COVID-19 patients have been equivocal.^59–65^ In a study, early blockade of IL-1β signaling by IL-1RA exhibited significant survival benefit in severe hyperinflammatory respiratory failure in COVID-19 patients.^59^ In another trial, high dosages of IL-1RA also showed clinical improvements in patients.^60^ In an open-label trial of anakinra in 120 patients, anakinra decreased risk of progression to severe respiratory failure by 70%.^64^ However, in mild-to-moderate COVID-19 disease, anakinra treatment did not improve outcomes.^63^ Our results in a controlled experimental model of SARS-CoV-2 infection and lung injury may provide insights into the variable clinical findings and identify patients most likely to benefit from IL-1RA treatment. We found that IL-1RA reversed the downregulation of VE-cadherin in lungs, thus restoring lung endothelial barrier function, preventing lung edema, and improving survival.

IL-1β is a key mediator released by activation of inflammasome NLRP3—a cytoplasmic protein complex mediating the production of mature proinflammatory cytokine IL-1β via the inflammatory caspase-1.^66^ This complex includes polymerization of ASC (apoptosis-associated speck-like protein containing a caspase recruitment domain) and recruitment and activation of caspase-1.^66^ While our previous study showed that IL-1β release followed the activation of NLRP3 inflammasome in bacterial ARDS and induced lung vascular hyperpermeability,^17,67^ the present study shows a SARS-CoV-2 infection induces similar changes. IL-1R antagonism prevented the IL-1β–mediated decrease in expression of the endothelial junctional protein VE-cadherin, which maintains the integrity of endothelial adherens junctions.^68^ The excessive generation of proinflammatory cytokines such as IL-1β during the cytokine storm described in severe SARS-CoV-2 infection^10–12^ likely leads to widespread VE-cadherin downregulation in lungs and thus explains the high incidence of pulmonary edema and respiratory failure in severe COVID-19 patients. The lung endothelium is especially vulnerable to inflammatory activation because it expresses higher levels of inflammatory genes than endothelial cells of other vascular beds,^69^ and the lung endothelium is also known to serve as a source of IL-1β in ARDS.^18^

Increasing evidence suggests that vascular endothelium is an important target tissue in response to SARS-CoV-2 infection.^5,39,70–73^ A highly debated issue is whether SARS-CoV-2 directly infects and impairs functions of the endothelium. Some previous reports^74–76^ showed some human endothelial cells have lower or nondetectable expression of ACE-2 and TMPRSS2 and are less susceptible to SARS-CoV-2 infection. However, none of these results were directly obtained from the primary hLMVECs, which were used in our current study. Consistent with our observations, Caccuri et al^73^ recently demonstrated a direct role of SARS-CoV-2 infection of hLMVECs, inducing vascular dysfunction during the early phases of infection by using an immunofluorescence assay and in situ RNA hybridization, as well as proteome analysis. The present study provides direct evidence that hLMVECs are susceptible to SARS-CoV-2 infection and there is comparable mRNA expression of ACE-2 and TMPRSS2 in hLMVECs and AT-II epithelial cell line-A549 as evaluated by 1-step RT-PCR and real-time PCR. Further, we show that SARS-CoV-2 infection in hLMVECs induced activation of NLRP3–caspase-1–IL-1β inflammatory signaling pathway and pyroptotic cell death in a dose-dependent manner. SARS-CoV-2 infection and entry to cells were also evident by the detection of SARS-CoV-2 spike protein in endothelial cell lysates. It has been recently shown that tissue-specific heterogeneity and plasticity of the endothelium is maintained during systemic in vivo inflammatory injury.^69^ It is possible that tissue-specific endothelial cultures display variable susceptibility to SARS-CoV-2 infection for their different expression abundance of SARS-CoV-2 receptors. Results from some groups concerning the infectability of human endothelial cultures might thus be due to age of cells or using endothelial derived from different organs, which may lack the capacity of viral uptake because of low or nonexistent expression of ACE-2 and TMPRSS2.

The present findings suggest a path forward for clinical trials deploying IL-1 receptor antagonism in COVID-19 by selecting the target patient population most likely to benefit from this therapy. NLRP3 inflammasome activation in circulating peripheral blood mononuclear cells mirrors the activation seen in the lung itself.^13^ Our results suggest that patients showing evidence of NLRP3 activation are likely to benefit from treatment with IL-1 receptor antagonism. A parallel approach would be patient selection based on evidence of endothelial injury, such as through measurement of circulating endothelial microparticles.^18^ By introducing such an approach, it would be possible to maximize the benefits of immunomodulation through blockade of the IL-1 receptor.

## Article Information

### Acknowledgments

We thank Dr Maria Swerdlov at the Histology Tissue Core for her support. A.B. Malik conceived the study. S. Xiong designed the research with A.B. Malik and J. Rehman, wrote the biosafety level 3 (BSL-3) protocols, performed experiments (Figures 1, 3, 4, and 5 and Figure I in the Data Supplement), and drafted the initial manuscript. L. Zhang and S. Xiong performed experiments (Figure 2) and analyzed data under the guidance of J. Rehman. J.M. Richner and J. Class provided SARS-CoV-2 (severe acute respiratory syndrome coronavirus 2) and BSL-3 training. A.B. Malik and J. Rehman directed the study and critically reviewed and finalized manuscript and revision with S. Xiong. All authors reviewed the manuscript and provided critical feedback.

### Sources of Funding

This work was supported, in part, by the National Institutes of Health grants P01-HL060678, T32-HL007829, R01-HL154538, R01-HL149300, R01-HL118068, R01-HL157489, and R01-HL152515, as well as by intramural funds of the University of Illinois, College of Medicine.

### Disclosures

S. Xiong, L. Zhang, J. Rehman, and A.B. Malik are coinventors on a patent application describing ACE-2 (angiotensin-converting enzyme 2) peptides, which prevent the entry of coronaviruses into cells. S. Xiong is now employed by Merck Research Laboratories. The work in this article does not involve the use of ACE-2 peptides and does not involve the use of products developed by Merck Research Laboratories. The other authors report no conflicts.

### Supplemental Materials

Major Resources Table

Data Supplement Figures I and II

## Supplementary Material


