# Supplementary figures and images for: Interleukin-1RA Mitigates SARS-CoV-2–Induced Inflammatory Lung Vascular Leakage and Mortality in Humanized K18-hACE-2 Mice
Source: Arterioscler Thromb Vasc Biol. 2021 Sep 9;41(11):2773–85. doi: 10.1161/ATVBAHA.121.316925 (PMC8545251; doi:10.1161/ATVBAHA.121.316925)

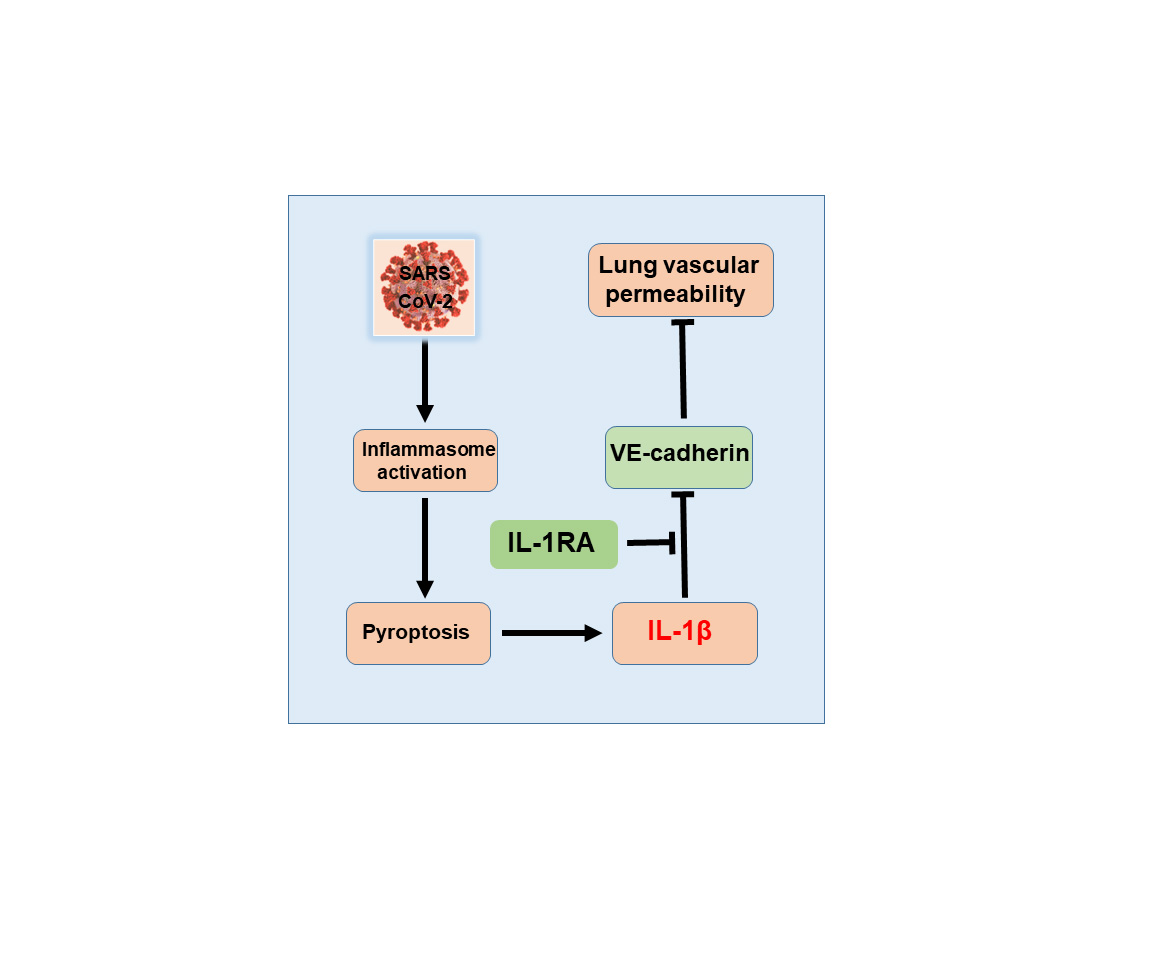

Supplement: Supplementary file 1 [file atv-41-2773-s001.jpg]
