# Supplementary material for: Interleukin-1RA Mitigates SARS-CoV-2–Induced Inflammatory Lung Vascular Leakage and Mortality in Humanized K18-hACE-2 Mice
Source: Arterioscler Thromb Vasc Biol. 2021 Sep 9;41(11):2773–85. doi: 10.1161/ATVBAHA.121.316925 (PMC8545251; doi:10.1161/ATVBAHA.121.316925)
Supplement: Supplementary file 2 [file atv-41-2773-s002.pdf]

SUPPLEMENTAL MATERIALS

Interleukin-1RA Mitigates SARS-CoV-2-Induced Inflammatory Lung Vascular Leakage and Mortality in Humanized K18-hACE-2 mice

Shiqin Xiong[1], Lianghui Zhang[1], Justin M. Richner[2], Jake Class[2], Jalees Rehman[1,3,#] and Asrar B. Malik[1,#]

[1]Department of Pharmacology and Regenerative Medicine, and the Center for Lung and Vascular Biology,

[2]Department of Microbiology and Immunology, [3]Division of Cardiology, Department of Medicine

The University of Illinois College of Medicine, Chicago, IL 60612, USA

Running Title: Covid 19 and Lung Vascular Injury

[#]Please address correspondence to:

Asrar B. Malik, PhD (abmalik@uic.edu) or Jalees Rehman, MD (jalees@uic.edu)

Department of Pharmacology & Regenerative Medicine

The University of Illinois College of Medicine

835 South Wolcott Avenue, Chicago, IL, 60612

Phone: (312) 996-7635

Fax: (312) 996-1225

Major Resources Table

Animals (in vivo studies)

Species	Vendor or Source	Background Strain	Sex	Persistent ID / URL
K18-hACE mice (strain#034860: B6.Cg-Tg(K18-CE2)2Prlmn/J)	The Jackson Laboratory	C57BL/6J	Male and female	034860 - B6.Cg-Tg(K18-ACE2)2Prlmn/J (jax.org)

Cultured Cells

Name	Vendor or Source	Sex (F, M, or unknown)	Persistent ID / URL
Vero E6	American Type Culture Collection (CRL-1586)	Unknown	VERO C1008 [Vero 76, clone E6, Vero E6] | ATCC
hLMVECs	LONZA (CC-2527)	Unknown	Human Lung Microvascular Endothelial Cells, HMVEC-L | Lonza
Human Lung Carcinoma Cells (A549) Expressing hACE-2	NR-53821, BEI Resources, NIAID, NIH	Unknown	BEI Reagent Search (beiresources.org)

Virus

Description	Source / Repository	Persistent ID / URL
2019n-CoV/USA_WA1/2019	NR-53821, BEI Resources, NIAID, NIH	BEI Reagent Search (beiresources.org)

Antibodies

Target antigen	Vendor or Source	Catalog #	Working concentration	Lot # (preferred but not required)	Persistent ID / URL
SARS-CoV-2 spike glycoprotein	BEI Resources	NR-94527	1:1000		BEI Reagent Search (beiresources.org)
NLRP3	AdipoGen	AG-20B-0014-C100	1:1000		anti-NLRP3/NALP3, mAb antibody (Cryo-2) - Order from Adipogen
Caspase-1 (p20)	AdipoGen	AG-20B-0042-C100	1:8000		Cleaved Mouse Caspase-1 (p20) Antibody (Casper-1) - Adipogen Life Science - For Inflammasome and Pyroptosis Research
Caspase-11	Novus Biologicals	NB-120-10454	1:8000		Caspase-11 Antibody (17D9) (NB120-10454): Novus Biologicals

Caspase-4	Santa Cruz Biotechnology	SC-56056	1:1000		Anti-caspase-4 Antibody (4B9) | SCBT - Santa Cruz Biotechnology
Caspase-5	Santa Cruz Biotechnology	SC-393346	1:500		Anti-caspase-5 p20 Antibody (H-2) | SCBT - Santa Cruz Biotechnology
IL-1β	R&D Systems	AF-401-NA	1:200		Mouse IL-1 beta /IL-1F2 Antibody AF-401-NA: R&D Systems (rndsystems.com)
CREB	Cell Signaling	9197	1:2000		CREB (48H2) Rabbit mAb | Cell Signaling Technology
VE-cadherin	Santa Cruz Biotechnology	SC-6458	1:1000		VE-cadherin Antibody (C-19) | SCBT - Santa Cruz Biotechnology
β-actin	Sigma	A-5316	1:10000		Monoclonal Anti-β-Actin antibody produced in mouse clone AC-74, ascites fluid (sigmaaldrich.com)

Figure I

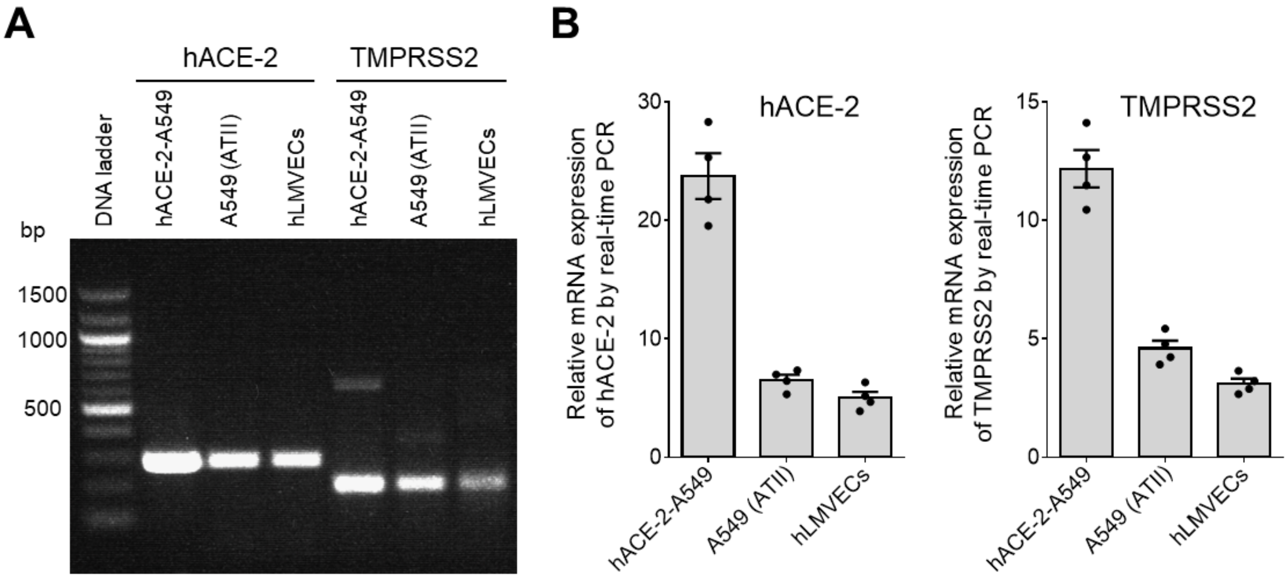

Fig I. mRNA expression abundance of ACE-2 and TMPRSS2 in hACE-2-stably expressed A549, A549 (ATII) and hLMVECs. mRNA expression levels of ACE-2 (NM_001371415.1) and TMPRSS2 (NM_001135099) in hACE-2-stably expressed A549 (ATII) epithelial, A549 (ATII) and hLMVECs were analyzed by one-step RT-PCR **(A)** and real-time PCR **(B)**, respectively. Relative expression was normalized by house-keeping gene GAPDH expression level.

Figure II

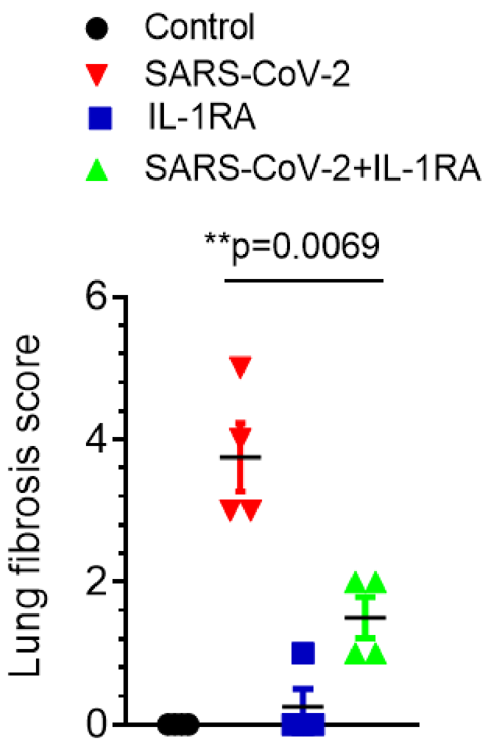

Fig II. IL-1RA mitigates SARS-CoV-2-induced lung fibrosis. K18-hACE-2 mice (2-month-old) were infected with a lethal dose of SARS-CoV-2 (1x105 p.f.u.). Mice also received the IL-1 receptor antagonist (IL-1RA) Anakinra (10 mg/kg/d) or vehicle by I.P. injection at 24 hours post infection and daily thereafter. Lung fibrosis was calculated using the Ashcroft method of analysis. Results are shown as mean ± SEM. **P< 0.01, two-tailed unpaired t test Tabular result.
